# Invisible contaminants and food security in former coal mining areas of Santa Catarina, Southern Brazil

**DOI:** 10.1186/s13002-020-00398-w

**Published:** 2020-08-14

**Authors:** Graziela Dias Blanco, Rafael Barbizan Sühs, Escarlet Brizola, Patrícia Figueiredo Corrêa, Mari Lucia Campos, Natalia Hanazaki

**Affiliations:** 1 Laboratório de Ecologia Humana e Etnobotânica, Departamento de Ecologia e Zoologia, Universidade Federal de Santa Catarina, Florianópolis, Santa Catarina Brazil; 2 Herbário Padre Dr. Raulino Reitz (CRI), Universidade do Extremo Sul Catarinense, Criciúma, Santa Catarina Brazil; 3 Laboratório de Análises Químicas do Solo e Calcário, Departamento de Solos e Recursos Naturais, Universidade do Estado de Santa Catarina, Lages, Santa Catarina Brazil

**Keywords:** Ethnoecology, Food security, Coal mining, Local communities

## Abstract

**Background:**

Mining activities have environmental impacts due to sediment movement and contamination of areas and may also pose risks to people’s food security. In Brazil, the majority of coal mining activities are in the south, in the Santa Catarina carboniferous region. In this region, previously mined areas contaminated with heavy metals frequently occur nearby inhabited zones. Mining is part of the daily lives of local communities, and its environmental impacts are visible in the landscape; however, plants with medicinal and food use from these areas can be still consumed. Heavy metals are contaminants that do not have odor, color, or taste, and are therefore difficult to detect. We aimed to verify whether people use plants from contaminated mine areas, and understand which factors can influence the use of these resources, even from areas visibly impacted.

**Methods:**

We conducted 195 semi-structured interviews with residents from 14 areas nearby abandoned mines in the main municipalities of the Santa Catarina carboniferous region. We asked each interviewee about the length of time they lived in the region, their perception of the quality of the environment, and what plant species were used and for what purpose. We constructed generalized multivariate linear models to verify which variables can affect the group of species mentioned and generalized linear models to verify which variables can affect the total number of citations. We estimated the frequency of citing species collected using the Smith index.

**Results:**

From all interviewees, 127 (65%) reported collecting plants for medicinal and food use, directly from contaminated mine areas. Long-term residents, as well as those who noticed more environmental changes (positive and negative), cited more plants used and had more detailed knowledge of plant use in their communities. When asked if they were aware of the possible contamination of mined areas, 85% said they knew about it. However, only 10% associated negative health effects with the use of plant species collected in contaminated mined areas.

**Conclusions:**

Our study demonstrates that people living nearby contaminated areas use and consume locally sourced plants, e.g., people know little about the danger of this contamination in their food and the risk of these contaminants to their health. These results also reveal a lack of information about contamination, as well as a lack of actions that include local communities in contaminated area restoration strategies. This situation poses a risk to the food security of the people living nearby former coal mining areas.

## Background

Around the world, contaminated areas have endangered people’s food security [1–3]. Among the sources of contamination, mining activities, such as coal mining, have caused public health concerns, due to the release of heavy metals in the mining process [1, 2, 4]. Although the impact level of metal toxicity depends on the concentration at which it is ingested, chronic exposure to relatively low levels of heavy metals may also cause adverse effects [5]. Heavy metals can bioaccumulate in the food chain; therefore, metals in the soil can be accumulated by plants that are consumed by humans, finally accumulating in humans [5, 6]. The effects of these elements in human health can be very diverse, depending on the metal and the exposure. For example, the ingestion of cadmium above the WHO-recommended levels can cause severe damage in the renal system, and excess of chromium can cause uterine cancer [7, 8].

Local communities are human groups, located in the same region and time, that develop a cultural identity and a unique relationship with the environment [9–11]. The interaction of local communities with the environment is directly related to their culture and the experiences and perceptions of past and present generations [10], and is reflected in the use of local resources and dietary habits [12, 13]. The study of heavy metal impacts on food security of local communities has gained prominence in regions such as China, related to urban growth in mined areas [1, 14], and northern Europe, related to increased mining activities and insecticide use in agriculture [15]. In Canada, there has been an increase in heavy metals in some foods used by indigenous communities [16]. In Latin America, studies with indigenous peoples and fishers have observed the presence of heavy metals in fish and plant resources consumed by local communities [17–20].

In Brazil, contamination of soil, plant, and fishery resources also poses a health risk to local communities [21, 22]. Coal mining in Brazil began in the late nineteenth century, and today, practically all coal produced in Brazil is mined in the southern area [23, 24]. Local communities began to settle in the neighborhoods of these mining areas since the decade of 1940, most of them comprised descendants of German, Italian, and Portuguese immigrants who work in mining [25, 26]. Before the arrival of European immigrants, the broad region was inhabited by Guarani and Xokleng Amerindians, with no other industrial or mining activity [27]. As a new mineral extraction area was discovered, the forest cover vegetation was removed and a settlement was developed in the surroundings, to serve the mining workforce [28]. Along the time, the major settlements gave rise to the urban areas of the region such as Criciúma and Lauro Muller [28]. Today, most inhabitants are descendants from those European immigrants and from the process of formation of a Brazilian identity which also mixed other cultures and ethnicities; groups of Amerindians remain in small groups, most of them restricted to very few indigenous territories [27]. The major period of coal mining was between 1975 and 1985, with an increase of these settlements; yet, coal mining is still one of the main economic activities together with agriculture [28, 29]. From the decade of 1980 onwards, the number of mining areas increased, but the number of local communities settled nearby these areas remain the same [30]. As a result of this historic process, some communities were settled very close to mines, or even partially on restored mined areas.

It is estimated that in the state of Santa Catarina alone, there are more than 6500 ha of abandoned areas contaminated by heavy metals from coal mining activities [31]. Due to diminishing profitability in the late twentieth century, some mined areas were abandoned while local communities developed in these locations. Even after decades of inactivity, abandoned mine areas are still contaminated by heavy metals [32] and may pose a risk to the food security of these communities. Some abandoned open-pit coal mines were restored according to each company’s practices, reconstructing the landscape and soil to create minimal conditions for vegetation development [33]. The restoration process consists basically of filling the pit with pyrite and covering this layer with another layer of clay soil, covering this sterile layer with clayey regolith, and putting back soil, followed by planting species for soil fixation [34]. However, this restoration process is usually deficient, especially due to the different mining processes employed by mining companies and the lack of inspection of mined areas by responsible authorities, resulting in contamination of the surface layers of the soil with coal residues [31, 34]. Some plant species can survive and even thrive in these contaminated sites [35–38] and can be bioindicators of contamination and useful for bioremediation, if they have bioaccumulation potential [35, 37]. Some of these species, however, also have medicinal or food use, and therefore may pose a risk to human health [39]. Nevertheless, few studies are investigating whether plant resources occurring in areas contaminated by heavy metals are being used by the local population [40].

People perceive and categorize changes in the landscape over time [41, 42]. The perception of changes in the landscape by local inhabitants (e.g., changes in species diversity and richness, and air pollution) assists in understanding the environmental consequences of impacts such as urbanization, deforestation, and mineral extraction [43, 44]. Generally, individuals living for longer and closer to the resources are those who have greater knowledge and use of the plant resources [42]. Coupled with this, women tend to have greater knowledge and use of medicinal plants, as they are usually responsible for early health care in several local communities [45–48]. Women are also more vulnerable to food security issues than men due to gender inequalities. Food security is the term used to define the right that all people, at all times, should have physical, social, and economic access to sufficient, safe, and nutritious food that meets their dietary needs and preferences for an active and healthy life [49]. Women often live in more unhealthy or contaminated areas than men, and, when they receive food from the government, it is of lower nutritional quality [49].

Considering the growing risk of contamination of plant resources [13, 18, 50, 51], we aimed to investigate whether local residents use plants obtained from contaminated mined areas and to understand which factors are related to plant use. We hypothesize that (1) the total number of species cited by interviewees will be affected by their residence time, sex, locality, perception of landscape change, and area type (i.e., either abandoned or partially restored) and (2) the group of cited species will be related to interviewees’ residence time, sex, locality and perception of landscape change, and area type (i.e., either abandoned or partially restored). We expect that women, older residents, residents neighboring restored mined areas, and residents who are unaware of the contamination will use and know more plant species.

## Methods

### Study area

**Fig. 1:**
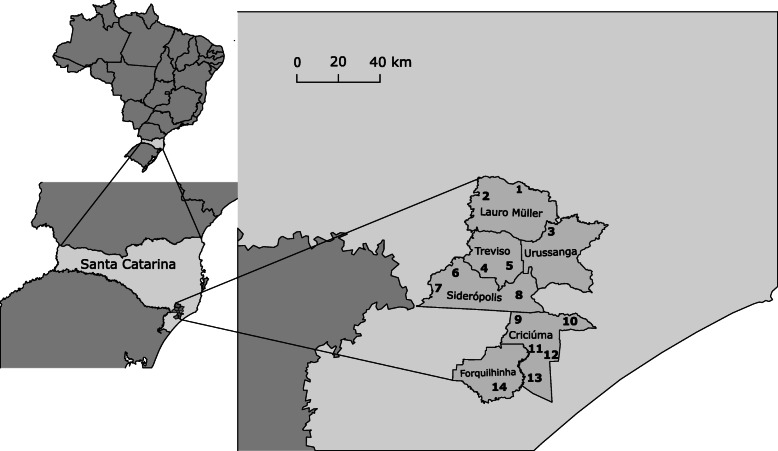
Study area. Each number corresponds to a mining community: 1, Barreiros; 2, Guaitá; 3, Rio Carvão; 4, Volta Redonda; 5, Rio Morozini; 6, Vila Funil; 8, Vila São Jorge; 9, Rio Fiorita; 10, Santa Luzia; 11, São Sebastião Alto; 12, Santa Augusta; 13, São Sebastião; and 14, Cidade Alta

The study was conducted in the municipalities of Criciuma, Forquilhinha, Siderópolis, Treviso, Urussanga, and Lauro Müller, in the state of Santa Catarina (Fig. 1), in the main coal mining region in southern Brazil. We selected 14 former coal mine areas, according to the following criteria: areas of at least 1 ha, which were abandoned for at least 30 years, with a history of heavy metal presence [32, 34, 52, 53], and with inhabited zones located at a maximum distance of 300 m from the deactivated mine areas. Some of these abandoned mine areas underwent an initial restoration process, which consisted of filling the pit with pyrite and covering this layer with another layer of clay soil (30–50 cm) over the disturbed mine soil. The mining communities in Vila Funil, Rio Carvão, Barreiros, Guaitá, Cidade Alta, Vila Visconde, and São Sebastião Alto are settled near abandoned mine areas, and the mining communities of Vila Sao Jorge, Rio Fiorita, Volta Redonda, Campo Morozini, Santa Luzia, Santa Augusta, and São Sebastião are adjacent to partially restored mine areas.

### Data collection

We conducted semi-structured interviews with residents of the communities located in inhabited areas near the mined areas, individually, between February and March 2018 (Table 1). To interview the residents individually when there was more than one adult at the time of the interview, we asked only one person to respond, and when possible, we moved to a more reserved place to conduct the interview. We visited every house in each community once and interviewed those who were keen to participate in the research and who agreed with the free informed consent terms. One limitation of this method is that our sampling is possibly skewed for people who stay in their homes more often, but as we intended to cover all mining areas in the region, we had to choose between a broad sampling effort and an in-depth sampling effort.

**Table 1:** General information of localities, total rural population of each municipality, total number of families per community living nearby mining areas, and number of interviews

Municipality	Total rural population of the municipality	Locality	No. of families per mining community	No. of interviews
Siderópolis	2944	Vila Funil	35	16
		Vila São Jorge	20	7
		Rio Fiorita	36	20
Lauro Muller	3261	Barreiros	27	16
		Guaitá	21	19
Criciúma	2678	Santa Luzia	37	8
		Vila Visconde	35	11
		São Sebastião Alto	25	8
		Santa Augusta	14	10
		São Sebastião	25	12
Treviso	1694	Volta Redonda	24	11
		Rio Morozini	26	16
Urussanga	8818	Rio Carvão	32	25
Forquilhinha	4122	Cidade Alta	23	16

Interview questions sought to understand: (1) whether plant species were collected or planted for consumption in areas contaminated by coal mining, (2) which were the main species collected and for what purpose, and (3) the interviewee’s perceptions of landscape changes and the impacts of mining. For each interviewee, the following variables were recorded: residence time, age, gender, locality, and their work relationship with the mining companies. To be sure of where the plant resources were obtained, the interviewee was asked for each species cited: whether they were collected from contaminated areas, collected in other areas, or planted in home gardens or other cultivated areas.

To analyze the perception of landscape changes, the interviewee was asked if they had observed any changes at the site since they began living there, and the responses were categorized a posteriori by the authors as (1) positive, e.g., positive changes have been observed over time in the landscape, such as an increase in the group of cited species; (2) neutral, e.g., no change was observed; and (3) negative, e.g., negative changes have been observed over time, such as in the group of cited species (Table 2). According to the interviewees, the positive aspects were mainly related to the mention of a good environment to live, with trees, birds, or a calm and safe place. Regarding the negative perception, the interviewees used the word “degradation” due to mining, referring to esthetically ugly places, air pollution, abandoned and careless areas, and areas without infrastructure for leisure, such as parks.

**Table 2:** Summary of the variables raised during the interviews and used in the GLM analysis as tested variables

Variable	Explanation
Perception	Observing environmental changes where you live.
	1 Positive—has observed positive changes over time in the landscape, such as increased plants and animals.
	2 Neutral—not observed any change.
	3 Negative—has observed negative changes over time in the landscape, such as species loss.
Locality	Local community.
Gender	Men and woman.
These areas	In which mined context the interviewee lives.
	Mined and abandoned area: these areas are visibly degraded and with exposed tailings.
	Mined and abandoned area partially restored: these are greener areas with soil covered by a layer of clay and grass.
Residence time	How many years have residents lived in this community.

They were also asked if they knew what the landscape looked like before mining, whether mining impacts have or had a negative impact on the health of residents, and whether they had been informed (either by public or private institutions) about contamination of the mined areas (the full questionnaire is accessible in Additional file 1). Whenever possible, we conducted guided tours to collect botanical samples of the cited species for identification (collector numbers GD Blanco 90-120, vouchers deposited at EAFM herbarium). This project was approved by the UFSC Human Research Ethics Committee (80660217.1.0000.0121) and registered at SisGen, the Brazilian System of Genetic Heritage and Associated Traditional Knowledge Management (AB9A76B). Prior to the interviews, the consent of each interviewee was obtained, and they signed a free informed consent form.

### Data analysis

We built multivariate generalized linear models (GLMmv) to verify which variables could affect the group of cited species and generalized linear models (GLM) to verify which variables could affect the total number of citations. For both analyses, we discarded information about plant species that were cited as cultivated only and used data from the species cited as being collected from mined areas. The explanatory variables for both set of models were residence time, gender, type of abandoned area (i.e., abandoned or partially restored), and the locality where the interviewee lives. However, locality and area type were never put together in the models, as both variables are related to geographic location, thus highly correlated. For both models, the Poisson distribution family was used. Model selection was based on the Akaike Information Criterion (AIC) and validated using graphical residual analysis. For data visualization, a principal coordinate analysis (PCoA) was performed. Analyses were performed in the R environment with packages mvabund [54] for GLMmv, MASS [55] for GLM, and visreg [56] and vegan [57] for visualization of effects. The variables tested are listed in Table 2. For multivariate analysis, singletons (plants cited only once) and doubletons (plants cited twice) were removed.

To analyze the importance of the plants mentioned in the interviews, we used their frequency of citations and the Smith salience index given by Σ(((*Li* − *Rj*)/Li)100)/*N*, where *Li* is the size of the free listing, *Rj* is the position (order) of the item in a given free list (*Li*), and *N* is the total number of free listings (or the number of interviewees) [50]. We also considered that the first item of a given list has *Rj* = 0. This index ranges from 0 to 1; species with a value equal to or close to 1 are the species with the highest salience, and species with values close to 0 are the least salient. After assessing the Smith salience index, we calculated if the values differed by chance using a Monte Carlo analysis, following Chaves et al. [58].

## Results

We interviewed 195 residents, with an average of 14 residents (± 5.4) per locality. The residents’ ages ranged from 15 to 86 years old, with an average age of 53 years (± 17.8). The majority of the residents (115 or 59%) have lived in the community for more than 20 years (± 12.1), and 50 residents (26%) have always lived in the area, with the rest coming from other parts of the state. However, no respondents resided in the region before the coal mining. Among the residents, 130 were women (68%) and 66 were men (32%). All of the men, and none of the women, either work or have worked for the mining companies. Collecting or planting species for medicinal and/or food use was cited by 176 residents (90%), and 127 residents (65%) collected plants directly from areas contaminated by mining.

All of the 176 planted or collected species were cited (Additional file 2), of which 83 species (47%) were classified as collected from the mined areas. From these, 18 (10%) species were obtained exclusively through the collection in the mined areas (Table 3). The main species obtained exclusively by collecting from mined areas were *Psidium guajava*, *Plinia cauliflora*, and *Eriobotrya japonica*. The main botanical families collected were Asteraceae and Lamiaceae, with 10 species (10%) each, and Myrtaceae and Fabaceae with 4 species (3.5%) each. For species collected in mined areas, 78% of residents cited medicinal uses, and 76% of residents cited food uses. The main use (54%) for medicinal species was for the treatment of digestive and infectious problems.

**Table 3:** Species cited exclusively as collected by 195 interviewees, number of citations per species, uses, and salience (Smith’s index)

Species	Smith index	Salience *p* value	No. of citations	Use
*Psidium guajava* L.	0.12	0.00	30	F
*Plinia cauliflora* (DC.) Kausel	0.09	0.00	25	F
*Psidium cattleianum* Sabine	0.07	0.00	18	F
*Morus* sp.	0.06	0.00	14	F
*Foeniculum vulgare* var. *azoricum* (Mill.) Holub	0.05	0.01	12	M
*Chelidonium majus* L.	0.01	0.04	7	M
*Bidens pilosa* L.	0.01	0.01	6	M
*Fragaria vesca* L.	0.01	0.02	4	F
*Justicia pectoralis* Jacq.	0.00	0.00	3	M
*Aristolochia esperanzae* Kuntze	0.00	0.00	3	M
*Achillea millefolium* L.	0.00	0.00	3	M
*Eriobotrya japonica* (Thunb.) Lindl.	0.00	0.00	21	F/M
*Equisetum giganteum* L.	0.02	0.28	16	M
*Passiflora edulis* Sims	0.03	0.42	12	F
*Inga edulis* Mart.	0.04	0.16	11	M
*Baccharis* spp.	0.01	0.08	9	M
*Campomanesia xanthocarpa* Mart. ex O. Berg	0.01	0.08	6	F
*Butia capitata* (Mart.) Becc.	0.01	0.06	4	F

*F* food; *M* medicinal

Smith’s salience index for species collected directly from mined areas varied between 0.12 and 0.01. Species with the highest salience and with significant results after Monte Carlo analysis were *Psidium guajava*, *Plinia cauliflora*, *Psidium cattleianum*, *Morus* sp., *Foeniculum vulgare* var. *azoricum*, *Chelidonium majus*, *Bidens pilosa*, *Fragaria vesca*, *Justicia pectoralis*, *Aristolochia esperanzae*, *Achillea millefolium*, and *Eriobotrya japonica* (Table 3).

When questioned whether they were aware of the presence of contamination in abandoned mine areas, 166 residents (85%) said they were. However, when asked about harm to the environment or their lives, only 19 residents (10%) reported some type of physical discomfort (i.e., stomachache and low blood pressure), when ingesting the species *Baccharis* spp., *Plectranthus barbatus*, *Solanum paniculatum*, *Arnica montana*, and *Achillea millefolium*. Regarding harm to the environment, the interviewees cited atmospheric pollution, due to the excessive dust released in the region from coal extraction. All residents moved to the area after the mining activity ended and did not see what the landscape looked like before mining; however, 172 residents (88%) said they had observed changes; 113 residents (58%) reported negative changes, i.e., forest loss, fewer animals, fewer plants (this type of response was classified as negative, a posteriori, by the authors); and 58 residents (30%) reported landscape improvements, i.e., more trees, more plants, cleaner air (this type of response was classified as positive, a posteriori, by the authors). Another issue confirmed by 147 of the residents (75%) was the lack of information from public agencies and mining companies, about the environmental impacts that mining may cause and possible contamination of plant resources. Duration of residence time, gender, perceived changes in the landscape (i.e., positive, neutral, negative), location, and type of abandoned area (i.e., either abandoned or partially restored) did not affect the group of species cited by respondents (Additional file 3).

**Fig. 2:**
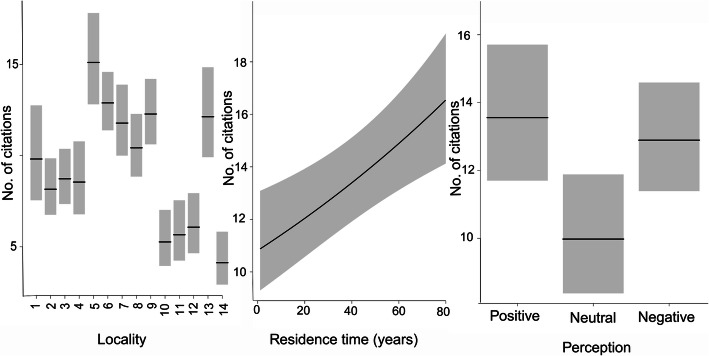
Graphical representation of the explanatory variables of the selected GLM model in relation to the number of species citations according to locality (1 to 14), residence time, and perception of landscape changes (i.e., 1, positive; 2, neutral; and 3, negative)

The same variables were analyzed concerning the number of species cited. The locality, perception of changes in the landscape, and duration of residence time significantly affected the total number of cited species. The GLM explained 27% of the variation in the total number of species used (Table 4, Fig. 2), of which locality explained 77%, perception of changes 15%, and residence time 8%. Longer-term residents and residents, who cited negative or positive landscape changes, cited more species collected than residents who had not observed landscape change over time. The locality also had a significant effect on the number of citations.

**Table 4:** Summary of models and variables tested with GLM

Mod.	Int.	Loc	Tip	Perc.	Gend.	Time	df	LogLik	AIC	Delta	Weight
22	2.074	+		+		0.004711	17	677.842	1389.7	0	0.276
30	2.089	+		+	+	0.004885	18	677.064	1390.1	0.44	0.221
6	2.231	+		+			16	683.686	1399.4	9.69	0.002
14	2.245	+		+	+		17	683.281	1400.6	10.88	0.001
18	1.93	+				0.006767	15	693.262	1416.5	26.84	0
26	1.941	+			+	0.006944	16	692.789	1417.6	27.89	0
2	2.148	+					14	706.557	1441.1	51.43	0
10	2.152	+			+		15	706.524	1443	53.36	0
31	2.206		+	+	+	0.005626	6	769.991	1552	162.3	0
23	2.19		+	+		0.005439	5	771.065	1552.1	162.5	0
29	2.163			+	+	0.005814	5	771.186	1552.4	162.7	0
21	2.145			+		0.005621	4	772.312	1552.6	162.9	0
17	2.016					0.006696	2	781.196	1566.4	176.7	0
25	2.029				+	0.006896	3	780.429	1566.9	177.2	0
19	2.045		+			0.006546	3	780.473	1566.9	177.3	0
27	2.057		+		+	0.006744	4	779.747	1567.5	177.8	0
7	2.366		+	+			4	779.804	1567.6	177.9	0
15	2.382		+	+	+		5	779.256	1568.5	178.8	0
5	2.318			+			3	781.676	1569.4	179.7	0
13	2.335			+	+		4	781.111	1570.2	180.5	0
3	2.264		+				2	794.521	1593	203.4	0
1	2.23						1	796.017	1594	204.4	0
11	2.271		+		+		3	794.403	1594.8	205.1	0
9	2.238				+		2	795.888	1595.8	206.1	0

*Mod.* model number, *Int.* intercept value, *Loc.* locality, *Tip* type of area (i.e., either abandoned or partially restored), *Perc.* perception of landscape changes, *Gend.* gender, *Time* residence time, *df* degrees of freedom, *LogLik* likely distribution of observed data, *AIC* Akaike Information Criterion, *Delta* difference of each model in relation to the model selected by AIC, *Weight* model weight

## Discussion

Residents living in mining communities near abandoned or partially restored coal mine areas are consuming plant species from these areas for food and medicinal purposes, which puts their food security at risk: almost a half of the plants cited were collected from mined areas, and 18 species were collected exclusively in these areas. The consumption of species that occur in mined areas was also reported in other regions of South America, as well as in the USA, Europe, India, and China [1, 14, 15, 17, 20, 59]. Some of these species have been studied for their potential to bioaccumulate heavy metals, such as *Psidium guajava*, *Morus* sp., *Baccharis crispa*, *Baccharis sarothroides*, *Mentha arvensis*, and *Cymbopogon flexuosus* which bioaccumulate Al, Fe, Si, S, Ca, and Zn [36–38, 60–62]. Location, duration of residence time, and perception of changes in the landscape were the main factors linked to citing more species obtained in contaminated areas.

Locality was the most important factor influencing the number of species cited (i.e., 77% of the 27% that explained the use of plant species from mined areas). Localities studied here are local communities settled originally to supply the coal mining economy with laborers to work in coal mines [23]. Over time, these mining communities developed bonds with their environment, learning about the plant resources available in each place, a behavior co-evolving with the available plant resources and influenced by expertise, and direct and continuous observation of the environment [63, 64]. This behavior is also influenced by transformations in the social and cultural structures, policy systems, and spiritual beliefs [63, 64]. Even though these environments present a low plant diversity [65], the mining communities adapted to use the available resources for their medicine and food purposes.

Besides, locality influenced species cited by the communities. This may be due to the high cultural diversity of people, including indigenous peoples such as Guarani and recent German and Italian immigrants [66, 67]. Santa Catarina, and other areas in the south and southeast of Brazil, is culturally heterogeneous, which may affect plant knowledge and use. The influence of different cultures and the mixture of knowledge are combined and integrated into the most recent generations [68]. This cultural influence may have a greater weight than, for example, the resource availability itself in the environment [68, 69]. The longer the time a person had resided in the area correlated with more species cited: older residents use a greater wealth of plant species, collected or planted, and they also perceive more changes in the landscape, both due to the length of time of living and learning in these environments [42, 64].

Residents who observed changes in the landscape, both positive and negative, cited more species than those who did not notice changes. Even when residents noted that there was a decrease in plant resources and negative landscape changes in areas contaminated by coal mining, they cited the use of plants collected in these areas for their food and medication. However, we emphasize that since the categorization as “positive,” “neutral,” or “negative” was made a posteriori by the authors, these results reveal a broad and simplified view of what is considered as a perception of improvement (i.e., positive) or loss (i.e., negative) in the environment. Similar observations are reported by Silvano and Begossi [22], who found that although fishermen knew about mercury contamination in fishery resources, they continued to consume this resource. As well as some residents noticing negative changes in the environment due to mining, 85% of residents said they know about the contamination of the mined areas; however, they still collect and use plant species from these areas. This apparent paradox may be due to contaminants such as heavy metals being invisible or due to psychological barriers [70, 71].

Invisible contaminants are those that cannot be detected by human sensory abilities, i.e., cannot be seen and do not exude odor, taste, or sound [70]. Since they are not perceived, these contaminants can be unwittingly consumed and, in the case of heavy metals, for example, can impact human health causing neurological damages and metabolic disorders [39, 70, 72]. Psychological barriers, on the other hand, are when people are aware of the environmental impacts but do not act emphatically against them [71, 73]. People tend to think of environmental impacts as futuristic and distant from their reality, associated with governments failing to present more effective strategies involving local people, and within a framework of contemporary cultural and social issues [71, 74]. The social understanding of risk, such as food security risk, is built on views and beliefs associated with the social and cultural forces of each society or community [75]. The construction of this perception goes through a comparison stage. For a mining community to perceive the risk to their own food security, it needs to see that a similar situation was identified as a risk, in another community that is culturally, socially, and historically similar to its own [75, 76].

No significant differences were observed in the group of species cited, and this can be due to the low diversity of plants available in mined areas. Few species can survive and develop in environments impacted by heavy metals [77]. The mining activity tends to result in more homogeneous environments, affecting the microbial and fungal diversity in the soil thereby affecting plant diversity [77, 78], revealing the threat to the biodiversity of these environments.

We did not find a difference between species cited by women and men. This homogeneous distribution of knowledge across genders was also observed in other studies [45, 47]. This may be related to the different social roles of each gender: men are the ones who work or worked in the mining areas, contributing to their knowledge of the plants that occur there. Even though women usually provide initial health care in communities and therefore have greater medicinal plant knowledge, in these localities, men have a greater knowledge of the mined areas and of species found there, which seems to balance the knowledge of plant uses [47, 48].

The use of plant species from areas contaminated by coal mining has also been observed in local communities in Europe, where these communities are among the most vulnerable to, and affected by, contamination of food resources [79]. Bolivia and other Latin American countries have warned of the risk to the food security of local communities near mined areas, primarily the consumption of fishery resources [19]. In China, foods that form the staple diet of local communities living near former coal mines (e.g., *Oryza* spp. and *Camellia sinensis*) are contaminated with heavy metals [80–83]. In Canada and the USA, rural and indigenous communities are twice as vulnerable to contamination of their food resources compared to the national average [84]. These communities have greater exposure to, and are in direct contact with, contaminating sources [13, 84], a situation similar to that faced by mining communities in southern Brazil.

The lack of food security due to the consumption of contaminated fishing resources has been reported in local fishing and river communities from the south, southeast, and northeast coasts of Brazil, as well as by indigenous Amazon communities [13, 22, 85]. In recent decades, the global return of incentives for coal extraction has raised concerns about the food security of local communities [32]. Coal is currently responsible for providing 29.6% of global energy needs and about 42% of all global electricity [32, 86]. The resurgence of coal mining may increase the contamination of areas previously mined for coal and add to the number of areas impacted by heavy metal contamination. In the far south of Brazil, children living in coal mined areas are at high risk of exposure leading to possible heavy metal poisoning [87]. For this reason, research that identifies whether there is use, and which species are being used for medicinal and food consumption in mined areas, is important to develop strategies aimed at guaranteeing the food security of mining communities.

## Conclusion

Traditional and local knowledge are important tools for identifying and locating areas and resources that can pose a risk to the food security of mining communities. Consumption of plant species collected from abandoned mine sites in southern Brazil, coupled with a lack of information, is a reality and a concern. Plant species that can potentially bioaccumulate do occur in these areas and are being used locally as food or therapeutic resources. This situation is aggravated by the fact that several contaminants from mining are invisible and because of psychological barriers to recognizing the risks related to the contamination of the environment. Given this scenario, it is necessary and urgent to inform the population about the risk of invisible contaminants to reduce their vulnerability to food insecurity, combined with studies that quantify the extent of heavy metal contamination in plant resources resulting from mining activity.

## Supplementary information

**Additional file 1.** Model of the applied interview [interviewer’s orientations between brackets].

**Additional file 2.** List of species cited as used by residents. C: Collected, P: Planted.

**Additional file 3.** PCoA showing that there was no difference in the set of species collected from mined areas between mining communities, perceptions of landscape changes (i.e. positive, neutral and negative), types of abandoned areas (i.e. either abandoned or partially restored) and gender (i.e. men and women).

## Data Availability

The datasets used and/or analyzed during the current study are available from the corresponding author on reasonable request.
