# Supplementary material for: Invisible contaminants and food security in former coal mining areas of Santa Catarina, Southern Brazil
Source: J Ethnobiol Ethnomed. 2020 Aug 14;16:44. doi: 10.1186/s13002-020-00398-w (PMC7427890; doi:10.1186/s13002-020-00398-w)
Supplement: Supplementary file 1 — Additional file 1. Model of the applied interview [interviewer’s orientations between brackets]. [file 13002_2020_398_MOESM1_ESM.pdf]

Interview - Ethnobotanical Project in the Coal Region

Interviewer name:
Date:
Interview number:
Interviewee name: [confidential information]
Age:
Gender: F ( ) M ( )
Location (Municipality, Street and House #):

1.How long have you lived in this place?
 1.1 What is your place of origin?
 1.2 Do you work or worked in coal mining?
2. Since you live here, what landscape changes have you observed?
 2.1Do you know or remember what the landscape looked like before mining?
 2.2 Do you know or has anyone ever talked to the community about the possible contamination of coal mined areas?
3. Do you collect medicinal plants that occur near your home? Yes ( ) No ( )
4. Do you collect food plants that occur near your home, such as fruits, vegetables or roots? Yes ( ) No ( )
5. Do you plant any medicinal plants in your home? Yes ( ) No ( )
6. Do you plant any food plants in your home such as vegetables or fruits? Yes ( ) No ( ) [if answer is NO for questions 3,4,5 and 6, skip to question 8]
7. What medicinal and food plants do you collect or plant, and for what? [M: Mined Area, MR: Restored Mined Area. Fill one line to each plant]
 Plant/Purpose/Which part do you use?/Harvesting or planting location/M or MR
8. Do you buy any medicinal plants? Yes ( ) No ( ) [if answer is NO skip to question 11]
9. Where do you buy medicinal plants?
10. What medicinal plants do you buy?
11. Have you ever felt uneasy or uncomfortable after having had tea made of collected or planted herbs/plants? Y ( ) N ( ) [if the answer is YES, write down which plant(s)]
12. What discomfort? [this question can be induced suggesting examples such as bellyache, headache]
13. Are you aware of mining areas near where you live? Do you know which mined areas exist near where you live? [Show the map with the mined areas and locate the interviewee where she/he lives. Write down the corresponding area numbers]
14. Do you know anyone who lives nearby this area who uses and collects medicinal plants? [write down the name and where the person lives]
