# Supplementary figures and images for: Invisible contaminants and food security in former coal mining areas of Santa Catarina, Southern Brazil
Source: J Ethnobiol Ethnomed. 2020 Aug 14;16:44. doi: 10.1186/s13002-020-00398-w (PMC7427890; doi:10.1186/s13002-020-00398-w)

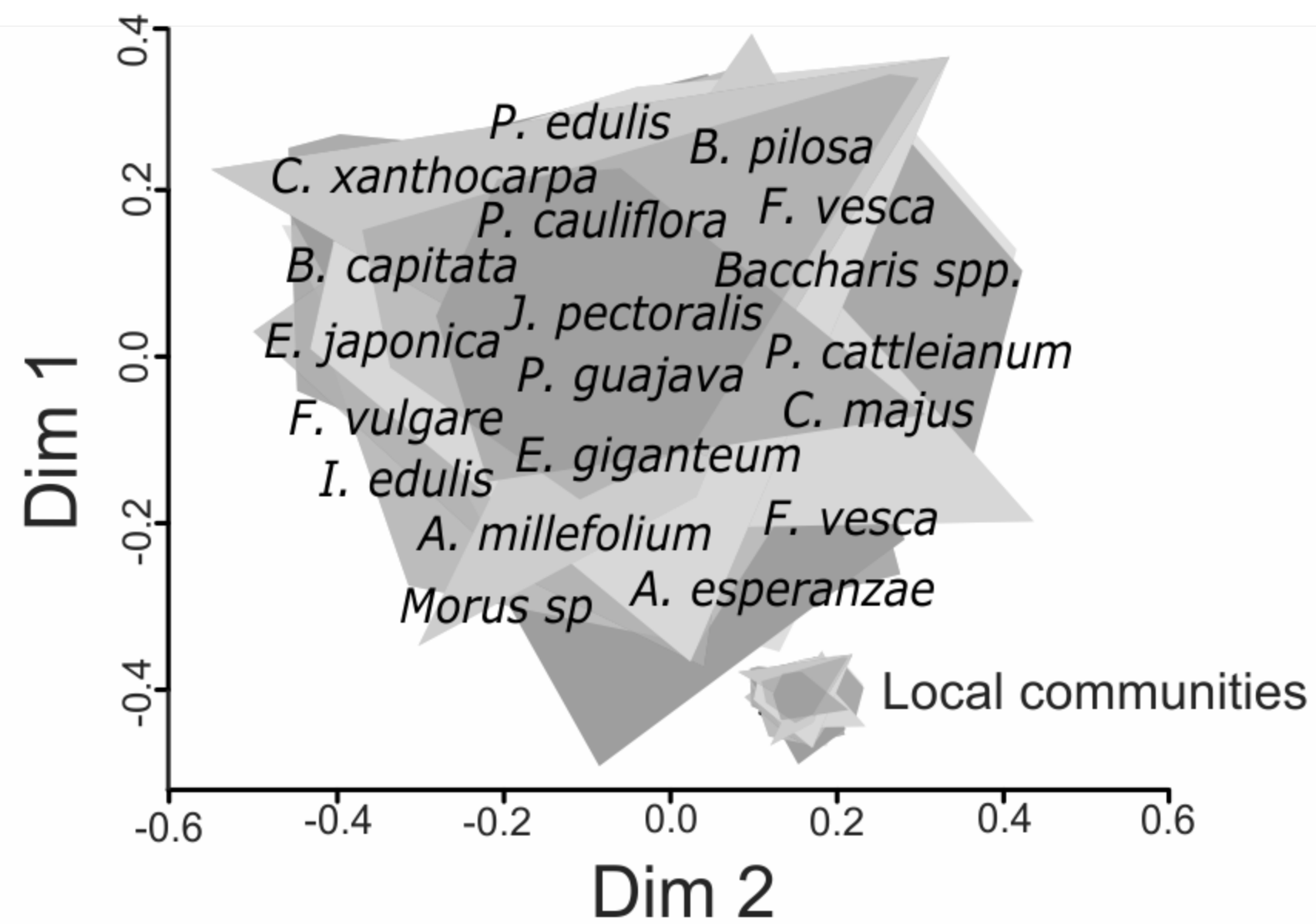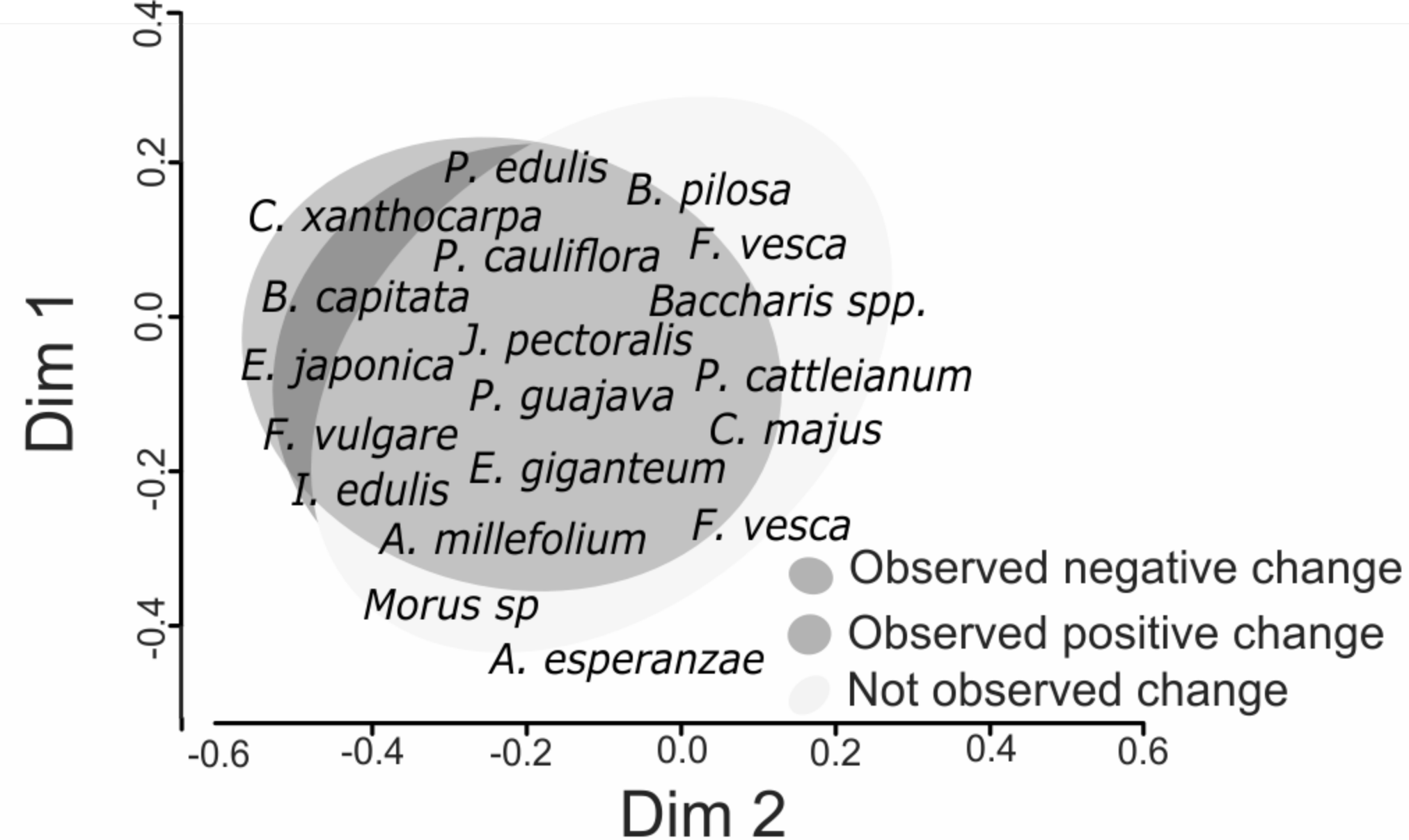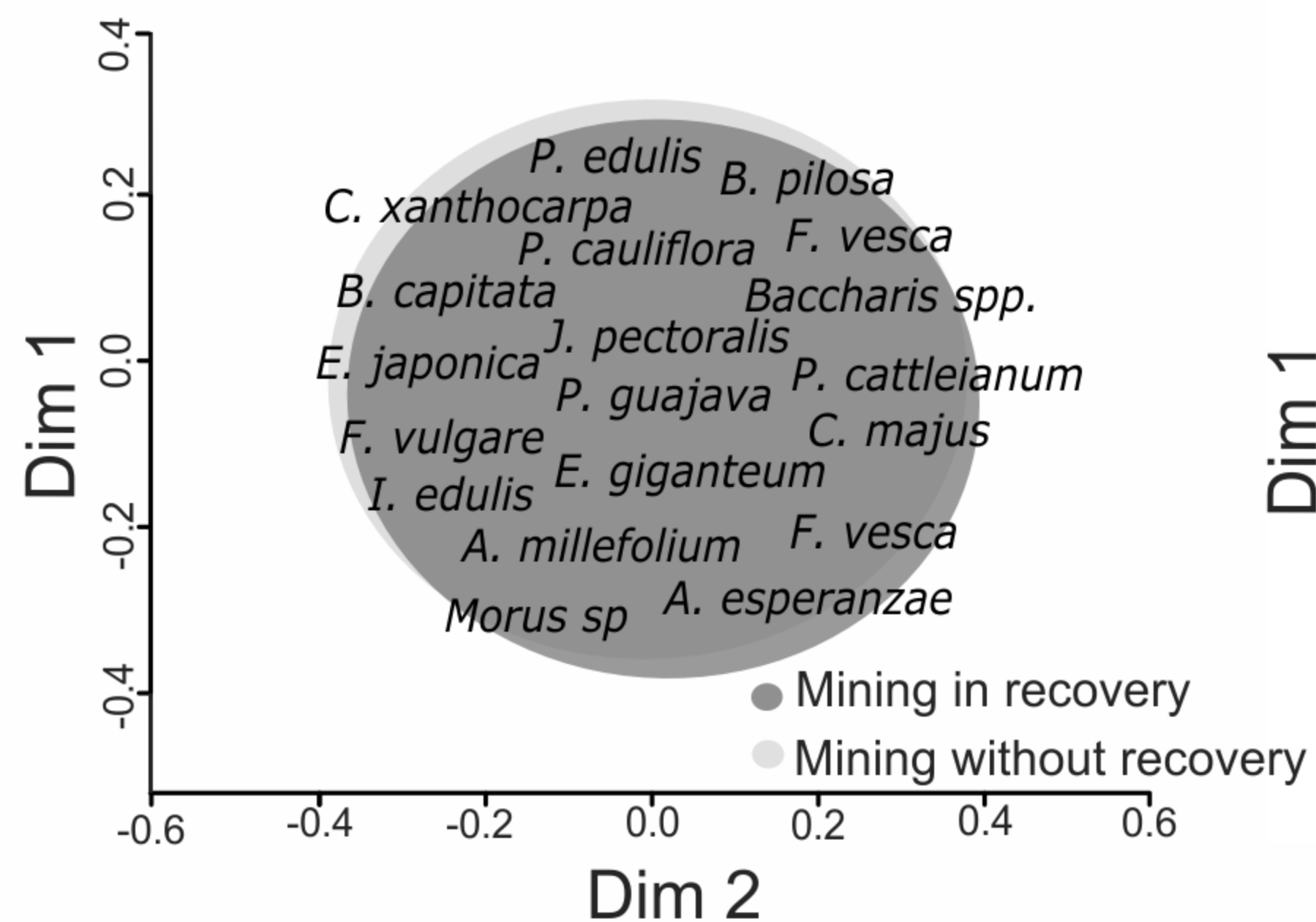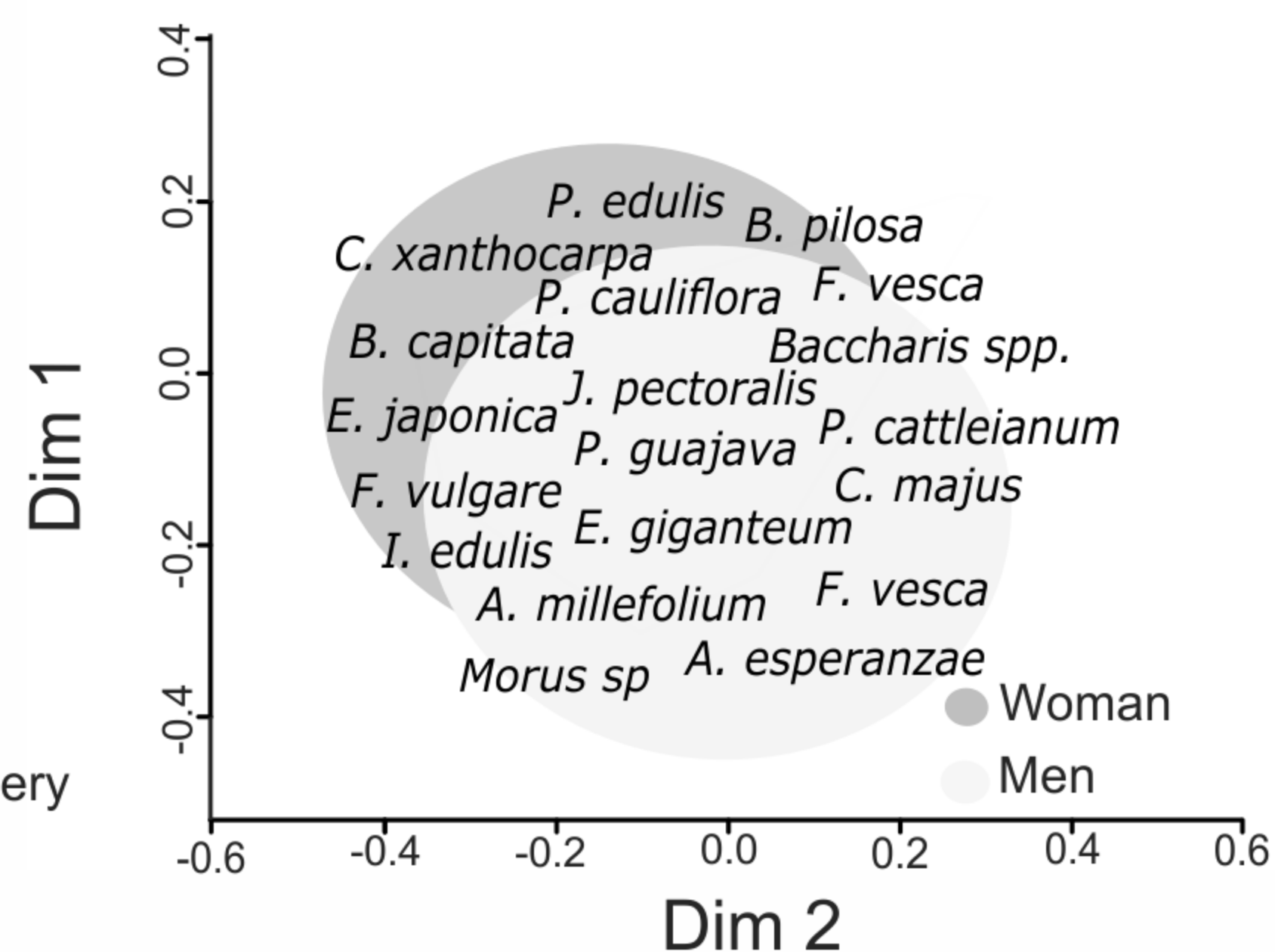

Supplement: Supplementary file 3 — Additional file 3. PCoA showing that there was no difference in the set of species collected from mined areas between mining communities, perceptions of landscape changes (i.e. positive, neutral and negative), types of abandoned areas (i.e. either abandoned or partially restored) and gender (i.e. men and women). [file 13002_2020_398_MOESM3_ESM.pdf]
